# Temperature-related indicators and risk of incident cardiovascular-kidney-metabolic multimorbidity in middle-aged and older adults: a longitudinal cohort study from CHARLS

**DOI:** 10.3389/fpubh.2026.1823025

**Published:** 2026-06-25

**Authors:** JiaXin Huang, Chang Ge, Xin He, JianChao Li

**Affiliations:** 1 Department of Extracorporeal Circulation, Fuwai Central China Cardiovascular Hospital, Zhengzhou, Henan, China; 2 Shengli Clinical Medical College, Fujian Medical University, Fuzhou, Fujian, China

**Keywords:** ageing, BMI, cardiovascular-kidney-metabolic multimorbidity, CHARLS, temperature

## Abstract

**Objective:**

To examine associations between temperature-related indicators and incident cardiovascular-kidney-metabolic (CKM) multimorbidity in middle-aged and older Chinese adults.

**Methods:**

We analyzed 4,665 CKM-free participants from the China Health and Retirement Longitudinal Study (2011–2018). CKM multimorbidity was defined as ≥2 conditions among cardiovascular disease, chronic kidney disease, and metabolic disorders. Exposures included 8-year mean temperature, persistent extreme cold events (≥5 consecutive days), and monthly temperature. Logistic regression, Cox models, a semi-Markov multistate model, and mediation analysis were used. Regional heterogeneity was tested via an interaction term between climate zone (cold/temperate/warm) and extreme cold events.

**Results:**

Over 7 years, 804 (17.23%) developed CKM multimorbidity. Each 1 °C increase in mean temperature was associated with 7.4% lower odds (OR = 0.926; 95% CI: 0.911–0.942), while each additional extreme cold event increased odds by 10.0% (OR = 1.100; 95% CI: 1.050–1.154). Higher monthly temperature reduced the hazard of transitioning from no CKM directly to complete CKM (HR = 0.905; 95% CI: 0.865–0.946), but not from partial to complete CKM. The effect of extreme cold events varied by climate zone: interaction was significant for temperate vs. cold zone (*p* = 0.045), with each event increasing odds by 15.6% in the temperate zone (OR = 1.156; 95% CI: 1.056–1.265). Protective effects of higher temperature were stronger in urban residents and those with depressive symptoms (*P*-interaction<0.05). BMI showed a potential mediation proportion of 14%, but this finding is exploratory and requires confirmation due to substantial missing BMI data.

**Conclusion:**

Long-term low temperature and persistent extreme cold events are associated with higher CKM multimorbidity risk, particularly in early disease transitions and with significant regional heterogeneity. Findings support targeted cold-region interventions such as improved housing insulation and early warning systems.

## Introduction

Population ageing is driving a global surge in cardiovascular-kidney-metabolic (CKM) multimorbidity, strictly defined as the co-occurrence of at least two conditions among cardiovascular disease, chronic kidney disease, and metabolic disorders (hypertension, type 2 diabetes, and dyslipidaemia) (1–3). Unlike isolated diseases, CKM multimorbidity arises from conserved pathogenic pathways—including chronic inflammation, oxidative stress, and renin-angiotensin-aldosterone system dysfunction—which cause synergistic systemic damage and substantially higher risks of clinical deterioration, hospitalisation, and mortality (4, 5). The burden is escalating worldwide, with low- and middle-income countries undergoing rapid epidemiological transitions; in China, geriatric CKM multimorbidity already dominates hospital admissions, accounts for over half of older adults’ healthcare expenditure, and threatens health-system sustainability (6–8). The American Heart Association’s 2023 scientific statement emphasises that CKM multimorbidity is not merely a random aggregation of separate diseases, but a synergistic syndrome driven by shared pathophysiological mechanisms (5). While conventional environmental pollutants contribute to individual CKM components, ambient temperature exerts distinct, immediate, and systemic effects on cardio-renal-metabolic homeostasis (9). Cold exposure triggers peripheral vasoconstriction and RAAS overactivation, increases cardiac afterload, reduces renal perfusion, and impairs insulin sensitivity, thereby accelerating sequential organ dysfunction and driving the transition from isolated disease to complex multimorbidity (10–15). Compounded by climate change, the increasing frequency and severity of extreme cold events pose a growing population-level threat (16–18).

Yet, epidemiological evidence remains largely confined to short-term associations with single CKM outcomes (19, 20). No longitudinal study has characterised how long-term cold exposure propels the dynamic progression of CKM multimorbidity across the full clinical spectrum—from disease-free status to single disorder, partial comorbidity, and advanced multimorbidity. Given that body mass index and depressive symptoms are plausible biological and behavioural mediators of cold-related metabolic and cardiovascular stress (21, 22), and that individual characteristics such as residential location and mental health status may modify susceptibility to temperature extremes (5, 18), we further examined their mediating and moderating roles in the temperature–CKM multimorbidity association. China’s vast geographical temperature gradient and rapidly ageing population provide an optimal setting to address this gap (23–25). Leveraging the nationally representative China Health and Retirement Longitudinal Study (CHARLS; 2011–2018) integrated with precise geocoded meteorological records (26–28), we systematically investigated the associations of long-term ambient temperature and persistent extreme cold exposure with the incidence and state transitions of CKM multimorbidity. Our findings will clarify the environmental etiology of CKM multimorbidity, identify temperature-vulnerable disease trajectories, and inform climate-adaptive public health policies to mitigate the growing multimorbidity burden in ageing populations worldwide.

## Methods

### Study design and population

This was a prospective cohort study based on data from CHARLS, a nationally representative longitudinal survey of middle-aged and older adults (≥45 years) in China. The study followed participants from the baseline wave (2011) to the 2018 follow-up wave, with intermediate assessments conducted in 2013 and 2015.

**Figure 1:**
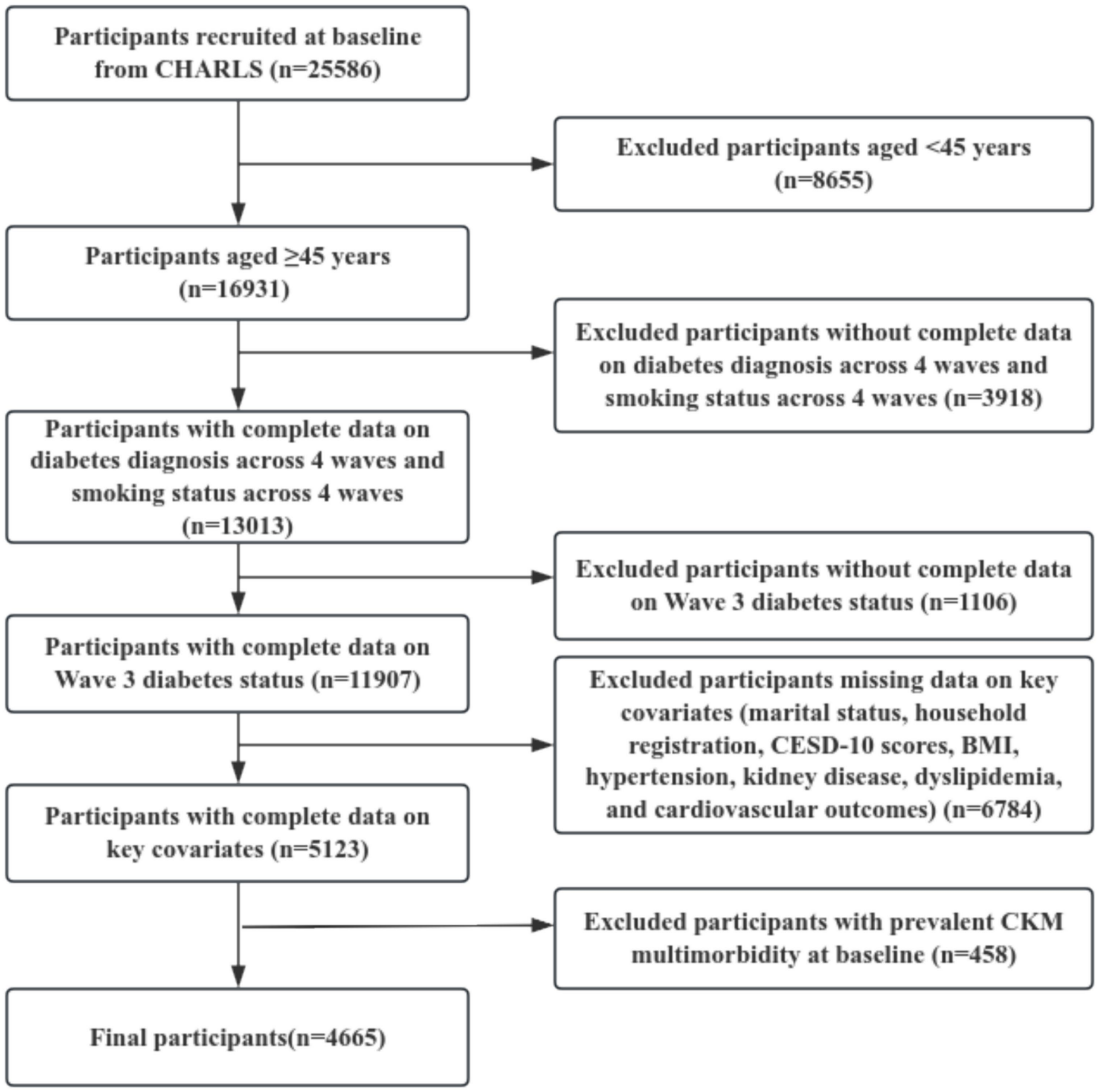
Flow chart of the cohort study.

Participants were eligible for inclusion if they met the following criteria: (1) completed the baseline CHARLS questionnaire with valid data on demographic characteristics, lifestyle factors, mental health, and clinical indicators; (2) had geocoded residential information available for matching with meteorological data; and (3) were free of CKM multimorbidity at baseline. CKM multimorbidity was defined as the co-occurrence of at least two of the three disease groups (cardiovascular disease, kidney disease, metabolic disease) at baseline. Participants with missing data on any key variable (including temperature exposure metrics, CKM outcome status, and covariates) were excluded from the final analysis. A flow chart of participant recruitment and exclusion is presented in Figure 1.

### Sensitivity analysis for selection Bias and missing data

Given the high proportion of participants excluded from the primary complete-case analysis (20,921 of 25,586 initial participants, 81.8%), a comprehensive sensitivity analysis was conducted to assess potential selection bias. First, missing data patterns across the initial sample were analyzed, revealing missing rates ranging from 0.03 to 46.72% for key variables (as shown in Supplementary Table S1). To enable a formal comparison between the analytic cohort and the full target population, multiple imputation was performed on the entire baseline dataset (*n =* 25,586) using the MissForest algorithm, a non-parametric method suitable for mixed data types. Subsequently, baseline characteristics were compared between the original analytic cohort (*n =* 4,665) and the imputed full cohort (*n =* 25,586) using standardized mean differences (*SMD*) (29). An *SMD* of <0.1 was considered indicative of a negligible difference, suggesting that the complete-case analysis was robust to selection bias arising from missing data (as shown in Supplementary Table S2).

The primary analytical cohort was based on the 2011–2018 CHARLS waves, considering substantial missing BMI data in the 2018 and 2020 surveys. To enhance result robustness, we further performed an extended sensitivity analysis covering the full 2011–2020 follow-up period, and the baseline characteristics of this extended cohort are presented in Supplementary Table S3.

### Data sources

1. CHARLS data

CHARLS data were collected using a multistage stratified sampling method, covering 150 counties/districts in 28 provinces across China. The survey included face-to-face interviews to collect information on: (1) Demographic characteristics: Age, sex, educational level (primary school or below/middle school or above), marital status (unmarried/divorced/widowed/married/cohabiting), and household registration type (rural/urban). (2) Lifestyle factors: Smoking status (never smoked/ever smoked) and alcohol consumption in the past year (yes/no). (3) Mental health: Depressive symptoms assessed using the 10-item Center for Epidemiologic Studies Depression Scale (CESD-10), with scores ranging from 0 to 30 (higher scores indicate more severe depressive symptoms). (4) Clinical indicators: Body mass index (BMI), calculated as weight (kg) divided by height squared (m^2^); self-reported history of stroke, heart disease, chronic kidney disease, hypertension, diabetes, and dyslipidemia.

1. Meteorological data

Meteorological data were obtained from the China Meteorological Administration (CMA), including daily observations of temperature (maximum, minimum, mean) from 2,400 national surface meteorological stations between 2011 and 2018. Each participant was matched to the nearest meteorological station based on residential geocoding data, with a maximum matching distance of 50 km to ensure data accuracy.

### Temperature-related exposure variables were derived as follows

1. *Long-term temperature indicators:* 8-year mean annual temperature, mean annual minimum temperature, and mean annual maximum temperature (2011–2018).
2. *Extreme temperature events:* persistent extreme cold events were defined as consecutive days with daily minimum temperature below the 5th percentile of the local temperature distribution; persistent extreme heat events were defined as consecutive days with daily maximum temperature above the 90th percentile of the local temperature distribution. The number of events with durations of ≥2, ≥3, ≥5, and ≥7 consecutive days was calculated for each participant.
3. *Seasonal temperature indicators:* mean temperature, mean minimum temperature, and mean maximum temperature for each season (Q1: January–March; Q2: April–June; Q3: July–September; Q4: October–December) during 2011–2018.
4. *Monthly temperature indicators:* mean monthly temperature for each follow-up month, used for survival analysis and multistate model analysis.

Such classification criteria for ambient temperature metrics and persistent extreme temperature events are consistent with previously validated environmental epidemiological methodologies (30, 31). This study focused primarily on persistent extreme cold events. Preliminary analysis showed that extreme heat events were not statistically associated with CKM multimorbidity in this national middle-aged and older cohort. Given the stronger mechanistic link between cold stress and cardio-renal-metabolic impairment, extreme cold was selected as the core research indicator. Baseline information on extreme heat was still presented for completeness (as shown in Table 1).

**Table 1:** Baseline characteristics of the study population stratified by incident CKM multimorbidity during 2011–2018 follow-up.

Variable	No CKM multimorbidity (*n =* 3,861)	With CKM multimorbidity (*n =* 804)	*P*-value
Demographic characteristics			
Age at Wave 1, years (median, IQR)	57.00 (51.00, 63.00)	59.00 (54.00, 64.00)	<0.001
Educational level, *n* (%)			0.871
Primary school or below	2,665 (69.02%)	552 (68.66%)	
Middle school or above	1,196 (30.98%)	252 (31.34%)	
Sex, *n* (%)			0.343
Male	1817 (47.06%)	363 (45.15%)	
Female	2044 (52.94%)	441 (54.85%)	
Marital status at Wave 1, *n* (%)			0.314
Unmarried/divorced/widowed	361 (9.35%)	85 (10.57%)	
Married/cohabiting	3,500 (90.65%)	719 (89.43%)	
Household registration type at Wave 1, *n* (%)			<0.001
Rural	3,363 (87.10%)	649 (80.72%)	
Urban	498 (12.90%)	155 (19.28%)	
Lifestyle and mental health			
Smoking status at Wave 1, *n* (%)			0.673
Never smoked	2,353 (60.94%)	497 (61.82%)	
Ever smoked	1,508 (39.06%)	307 (38.18%)	
Alcohol consumption in past year at Wave 1, *n* (%)			0.081
No	2,544 (65.89%)	556 (69.15%)	
Yes	1,317 (34.11%)	248 (30.85%)	
CESD-10 score at Wave 1 (median, IQR)	7.00 (3.00, 11.00)	8.00 (4.00, 13.00)	<0.001
Baseline clinical indicator			
Body mass index (BMI) at Wave 1, kg/m^2^ (median, IQR)	22.84 (20.60, 25.40)	23.94 (21.52, 26.75)	<0.001
Stroke diagnosis at Wave 1, *n* (%)			0.084
No	3,841 (99.48%)	795 (98.88%)	
Yes	20 (0.52%)	9 (1.12%)	
Temperature-Related Indicators (2011–2018)			
Mean annual temperature, °C (median, IQR)	16.88 (13.97, 18.50)	15.74 (11.95, 17.89)	<0.001
Minimum annual temperature, °C (median, IQR)	−5.85 (−12.67, −0.74)	−7.68 (−15.99, −2.83)	<0.001
Maximum annual temperature, °C (median, IQR)	31.55 (30.84, 32.81)	31.50 (30.78, 32.93)	0.131
Persistent extreme cold events, *n* (median, IQR)			
≥ 2 days	30.00 (26.00, 32.00)	30.00 (26.00, 32.00)	0.393
≥ 3 days	19.00 (18.00, 21.00)	20.00 (18.00, 21.00)	0.222
≥ 5 days	10.00 (9.00, 11.00)	11.00 (9.00, 12.00)	<0.001
≥ 7 days	5.00 (4.00, 7.00)	5.00 (4.00, 7.00)	0.019
Persistent extreme heat events, *n* (median, IQR)			
≥ 2 days	28.00 (25.00, 34.00)	29.00 (25.00, 33.00)	0.597
≥ 3 days	18.00 (15.00, 21.00)	18.00 (16.00, 21.00)	0.272
≥ 5 days	8.00 (6.00, 9.00)	8.00 (6.00, 9.00)	0.088
≥ 7 days	4.00 (3.00, 6.00)	4.00 (3.00, 6.00)	0.135
Seasonal mean temperature, °C (median, IQR)			
Q1 (Jan–Mar): Mean	7.78 (2.89, 10.30)	5.97 (0.38, 9.43)	<0.001
Q1 (Jan–Mar): Minimum mean	−1.27 (−6.81, 2.96)	−3.47 (−10.33, 0.90)	<0.001
Q1 (Jan–Mar): Maximum mean	18.79 (16.92, 20.29)	18.41 (15.44, 19.58)	<0.001
Q2 (Apr–Jun): Mean	21.50 (19.77, 22.61)	21.17 (18.56, 22.24)	<0.001
Q2 (Apr–Jun): Minimum mean	10.11 (7.29, 13.23)	8.74 (6.20, 11.99)	<0.001
Q2 (Apr–Jun): Maximum mean	29.13 (27.56, 30.05)	28.94 (26.87, 30.05)	0.196
Q3 (Jul–Sep): Mean	25.25 (23.24, 26.97)	24.91 (22.32, 26.48)	<0.001
Q3 (Jul–Sep): Minimum mean	17.72 (15.21, 19.06)	16.58 (12.91, 18.11)	<0.001
Q3 (Jul–Sep): Maximum mean	30.91 (28.88, 31.76)	30.79 (28.33, 31.89)	0.308
Q4 (Oct–Dec): Mean	12.20 (7.82, 14.55)	10.62 (4.47, 13.65)	<0.001
Q4 (Oct–Dec): Minimum mean	0.83 (−4.81, 4.88)	−1.23 (−7.99, 3.05)	<0.001
Q4 (Oct–Dec): Maximum mean	22.24 (19.88, 24.24)	21.20 (18.46, 22.88)	<0.001

CKM multimorbidity: Coexistence of at least 2 conditions among cardiovascular disease, kidney disease, and metabolic disease (hypertension, diabetes, dyslipidemia) during the 2011–2018 follow-up; Continuous variables are presented as median (interquartile range, IQR) due to non-normal distribution; categorical variables are presented as frequency (percentage); Statistical analyses: Categorical variables were compared using the *χ*^2^ test; continuous variables were compared using the Mann–Whitney U test; CESD-10: 10-item Center for Epidemiologic Studies Depression Scale; A two-tailed *p <* 0.05 was considered statistically significant; Extreme temperature events were defined based on local climate thresholds (consistent with the study’s retrospective analysis protocol for 2011–2018 temperature data).

### Variable definitions

#### Outcome variable

The primary outcome was incident CKM multimorbidity during the 2011–2018 follow-up. CKM multimorbidity was defined as the co-occurrence of at least two of the following three disease groups, based on self-reported physician diagnoses during follow-up: (1) Cardiovascular disease (CVD): Heart disease or stroke. (2) Kidney disease (KD): Chronic kidney disease. (3) Metabolic disease (MD): Hypertension, diabetes, or dyslipidemia. (4) For multistate model analysis, four subtypes of CKM multimorbidity were further defined: CK multimorbidity: Co-occurrence of CVD and KD; CM multimorbidity: Co-occurrence of CVD and MD; KM multimorbidity: Co-occurrence of KD and MD; Complete CKM multimorbidity: Co-occurrence of CVD, KD, and MD.

The time-to-event was defined as the interval from the baseline survey (2011) to the first diagnosis of CKM multimorbidity or the last follow-up (2018), whichever came first.

#### Exposure variables

Temperature-related exposure variables were categorized as continuous variables (per 1 °C increase) and categorical variables (quartiles [Q1–Q4], with Q1 as the reference group) to assess linear and non-linear associations with incident CKM multimorbidity, respectively.

#### Covariates

Covariates were selected based on prior epidemiological evidence and potential confounding effects, including: (1) Demographic factors: Age, sex, educational level (categorized as middle school education or above versus illiteracy), marital status (grouped as married/cohabiting versus all other statuses), and household registration type; (2) Lifestyle factors: Smoking status and alcohol consumption (defined as drinking versus non-drinking based on alcohol consumption within the past year at the time of survey); (3) Mental health factor: CESD-10 score; and (4) Clinical factor: BMI. Considering the lack of direct indicators for air pollution, diet and physical activity, we included residential location and household registration type as proxy variables to minimize residual confounding.

#### Mediator variables

BMI and CESD-10 score were selected as mediator variables to explore the mediating pathways underlying the association between temperature exposure and incident CKM multimorbidity, based on their biological plausibility (e.g., cold exposure may affect BMI via energy metabolism, and temperature may influence depressive symptoms through environmental stress). BMI was categorized into overweight (≥25 kg/m^2^) and normal weight (<25 kg/m^2^) groups, while CESD-10 scores were classified into depression (≥12 points) and normal (<12 points) groups for mediation analysis (29, 30).

### Statistical analyses

All statistical analyses were performed using R software (version 4.3.1; R Foundation for Statistical Computing, Vienna, Austria). A two-tailed *p* < 0.05 was considered statistically significant.

#### Descriptive statistics

Baseline characteristics of the study population were stratified by incident CKM multimorbidity status (yes/no). Continuous variables with non-normal distributions (assessed via the Shapiro–Wilk test) were presented as median (interquartile range [IQR]) and compared between groups using the Mann–Whitney U test. Categorical variables were presented as frequency (percentage) and compared using the *χ*^2^ test. The results are summarized in Table 1.

#### Association between temperature exposure and incident CKM multimorbidity

*Logistic regression analysis*: To evaluate the association between 8-year temperature indicators and incident CKM multimorbidity, we constructed three logistic regression models: Model 1 (unadjusted): No covariates included; Model 2: Adjusted for age, sex, and BMI; Model 3 (fully adjusted): Adjusted for all covariates (demographic factors, lifestyle factors, mental health, and clinical factors).

Odds ratios (ORs) and 95% confidence intervals (CIs) were calculated for both continuous (per 1 °C increase) and categorical (quartiles) exposure variables. The *p* value for trend (*P* for trend) was computed by assigning ordinal values to quartiles and treating them as continuous variables in the regression models. The results are presented in Table 2.

**Table 2:** Association between temperature indicators and incident CKM multimorbidity: ORs (95% CIs) and *P* for trend.

Exposure variable	Model 1 (unadjusted)	Model 2 (adjusted for age, sex, BMI)	Model 3 (adjusted for all covariates)
8-year mean temperature (°C)			
Q1 (Median: 12.89 °C, Reference)	–	–	–
Q2 (Median: 15.01 °C)	0.779 (0.638–0.952)	0.763 (0.624–0.934)	0.766 (0.625–0.938)
Q3 (Median: 17.79 °C)	0.616 (0.500–0.759)	0.586 (0.475–0.724)	0.590 (0.476–0.730)
Q4 (Median: 19.70 °C)	0.438 (0.350–0.549)	0.412 (0.328–0.517)	0.432 (0.343–0.544)
*P* for trend	0.015	0.009	0.01
8-year mean temperature (per 1°C)	0.926 (0.911–0.942)	0.922 (0.907–0.938)	0.926 (0.911–0.942)
*P*-value (continuous)	<0.001	<0.001	<0.001
8-year minimum temperature (°C)			
Q1 (Median: −11.25 °C, Reference)	–	–	–
Q2 (Median: −7.99 °C)	0.792 (0.648–0.967)	0.772 (0.631–0.944)	0.793 (0.648–0.972)
Q3 (Median: −3.66 °C)	0.599 (0.485–0.739)	0.578 (0.468–0.715)	0.573 (0.462–0.709)
Q4 (Median: 1.39 °C)	0.455 (0.364–0.569)	0.429 (0.343–0.538)	0.446 (0.354–0.561)
*P* for trend	0.022	0.012	0.025
8-year minimum temperature (per 1°C)	0.962 (0.953–0.970)	0.960 (0.952–0.968)	0.962 (0.953–0.970)
*P-*value (continuous)	<0.001	<0.001	<0.001
Persistent extreme cold events (≥5 days)			
Q1 (Median: 9.0, Reference)	–	–	–
Q2 (Median: 10.0)	0.993 (0.792–1.246)	0.996 (0.793–1.250)	1.020 (0.811–1.283)
Q3 (Median: 11.0)	1.323 (1.065–1.643)	1.360 (1.094–1.692)	1.349 (1.083–1.681)
Q4 (Median: 12.0)	1.388 (1.119–1.722)	1.422 (1.145–1.766)	1.383 (1.111–1.721)
*P* for trend	0.012	0.002	0.004
Persistent extreme cold events (≥5 days, per 1 event increase)	1.100 (1.050–1.154)	1.113 (1.062–1.166)	1.100 (1.050–1.154)
*P-*value (continuous)	<0.001	<0.001	<0.001

CKM, cardiovascular-kidney-metabolic multimorbidity; OR, odds ratio; CI, confidence interval; BMI, body mass index. Model 1 was unadjusted. Model 2 was adjusted for age, sex, and BMI. Model 3 was fully adjusted for all covariates including demographic factors, lifestyle factors, mental health (CESD-10 score), and clinical factors. Temperature variables were analyzed as continuous (per 1°C increase) and categorical (quartiles, with Q1 as the reference group). P for trend was calculated by assigning ordinal values to quartiles and treating them as continuous variables. The median follow-up duration was 7 years with total sample size *n =* 4,665. Statistical significance was defined as p < 0.05.

Restricted cubic spline (RCS) logistic regression: To explore the non-linear dose–response relationship between temperature indicators (mean temperature, minimum temperature, persistent extreme cold events ≥5 days) and incident CKM multimorbidity, RCS analysis with three knots (at the 10th, 50th, and 90th percentiles) was performed, adjusting for all covariates. The knot locations were selected based on standard recommendations for environmental epidemiological studies, which suggest placing knots at the 10th, 50th, and 90th percentiles to balance model flexibility and overfitting while avoiding instability at the extremes (31, 32).

#### Survival analysis

Cox proportional hazards regression analysis: To assess the association between monthly mean temperature and the risk of incident CKM multimorbidity, a Cox proportional hazards regression model was used, with follow-up time as the time scale. The proportional hazards assumption was verified using Schoenfeld residuals. The detailed results of Schoenfeld residual tests are presented in Supplementary Table S4. Hazard ratios (HRs) and 95% CIs were calculated after adjusting for all covariates. The results are shown in Table 3.

**Table 3:** Cox proportional hazards regression analysis of the association between monthly mean temperature and risk of new-onset CKM multimorbidity.

variable category	Variable name	Regression coefficient (*β*)	Standard error (SE)	Z statistic	*P*-value	Hazard ratio (HR)	95% CI for HR (lower–upper)
Core Exposure Variable	Monthly mean temperature (per 1 °C increase, centered)	−0.031	0.007	−4.791	<0.001	0.969	0.957–0.982
Covariates	Current age	0.016	0.006	2.521	0.012	1.016	1.004–1.028
	Sex	−0.104	0.147	−0.707	0.480	0.901	0.676–1.202
	Educational level	−0.153	0.118	−1.295	0.195	0.858	0.681–1.082
	Household registration type	0.196	0.131	1.501	0.133	1.217	0.942–1.571
	Marital status	0.163	0.149	1.092	0.275	1.177	0.879–1.576
	Smoking status	−0.015	0.138	−0.106	0.916	0.986	0.751–1.292
	Alcohol consumption status	−0.044	0.110	−0.395	0.693	0.957	0.772–1.188
	CESD depression scale score	0.051	0.007	7.448	<0.001	1.052	1.038–1.067

CKM, cardiovascular-kidney-metabolic multimorbidity; HR, hazard ratio; CI, confidence interval; CESD, Center for Epidemiologic Studies Depression Scale. Monthly mean temperature was centered at its mean value in the analysis. The model adjusted for all covariates listed in the table. The median follow-up duration was 7 years with total sample size *n =* 4,665. Statistical significance was defined as *P* < 0.05. For covariate coding, male was the reference group for sex. Educational level was categorized as middle school or above versus primary school or below. Marital status was grouped as married/cohabiting versus other statuses. Household registration type compared urban versus rural. Smoking status compared ever smoked versus never smoked. Alcohol consumption was defined as yes (consumed alcohol in past year) versus no.

**Figure 2:**
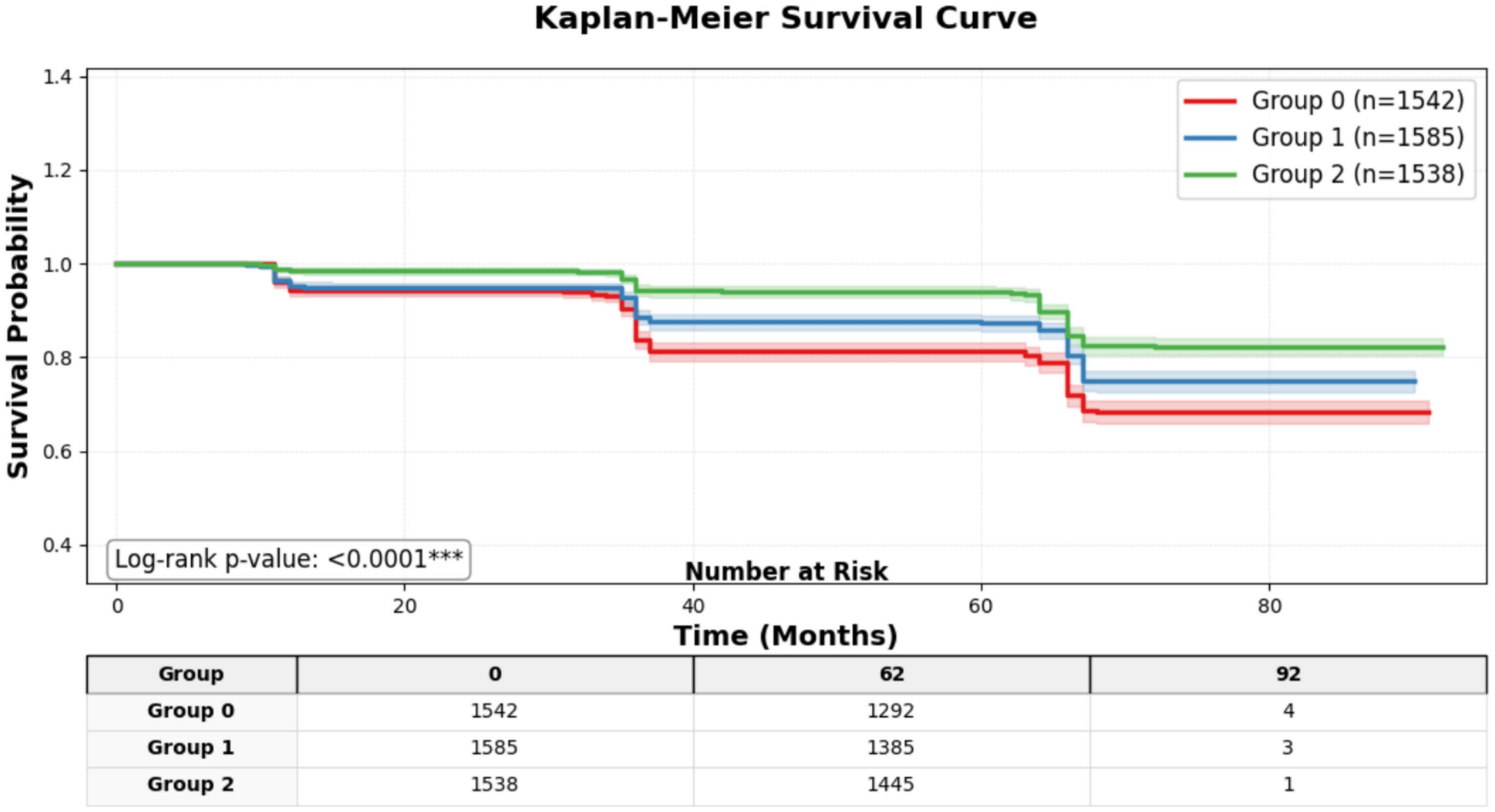
Kaplan–Meier curves for the incidence-free survival of CKM multimorbidity by monthly mean temperature groups. The Kaplan–Meier method was used to estimate the incidence-free survival rate of CKM multimorbidity across groups; log-rank test was used to compare survival differences among groups, with a statistical significance of ***p < 0.0001; the “Number at Risk” table below the curve shows the number of participants at risk of CKM multimorbidity at key follow-up time points (0, 62, and 92 months); the *y*-axis represents the incidence-free survival rate of new-onset CKM multimorbidity (range: 0–1), and the *x*-axis represents follow-up time (units: months).

Kaplan–Meier survival analysis: The incidence-free survival curves of CKM multimorbidity were plotted by monthly mean temperature groups. The log-rank test was used to compare survival differences between groups. The number of participants at risk at key follow-up time points was presented below the survival curves. The results are displayed in Figure 2.

#### Multistate model analysis

A semi-Markov multistate model was used to explore the relationships between monthly mean temperature and transition risks across five CKM health states (0 = no CKM, 1 = CK multimorbidity, 2 = CM multimorbidity, 3 = KM multimorbidity, 4 = complete CKM multimorbidity).

Forward progressive transitions were defined in this multistate structure, while reverse transitions were not allowed. Time-varying monthly temperature exposure and core baseline demographic, lifestyle and socioeconomic covariates were adjusted in all transition models. The semi-Markov assumption was satisfied via state-stratified estimation, and model convergence and goodness-of-fit were assessed to guarantee statistical validity.

**Figure 3:**
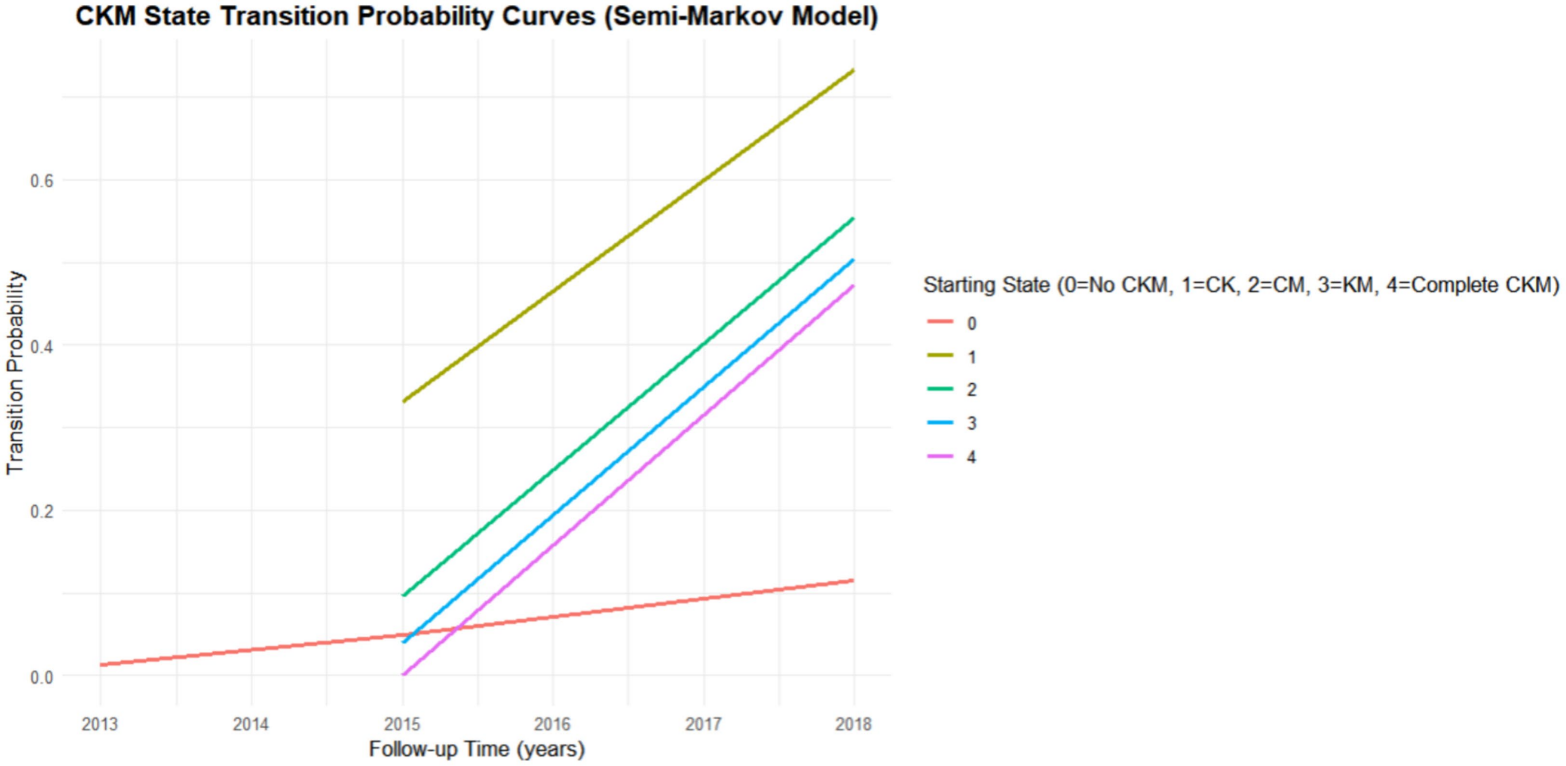
Temporal trends of CKM state transition probabilities based on the semi-Markov model (CHARLS, 2011–2018). X-axis: Follow-up time (years), ranging from 2013 to 2018 (derived from CHARLS Waves 2–4, with 2011 as baseline); Y-axis: State transition probability (range: 0–0.6), representing the likelihood of transitioning to or remaining in each CKM state at a given follow-up time. 0 = No CKM (no cardiovascular, kidney, or metabolic disease); 1 = CK multimorbidity (coexistence of cardiovascular and kidney diseases); 2 = CM multimorbidity (coexistence of cardiovascular and metabolic diseases); 3 = KM multimorbidity (coexistence of kidney and metabolic diseases); 4 = Complete CKM multimorbidity (coexistence of cardiovascular, kidney, and metabolic diseases).

Transition HRs and 95% CIs were estimated for each pathway. Line graphs were generated to illustrate temporal trends in state transition probabilities. The main results of the multistate analysis are shown in Table 4 and Figure 3.

**Table 4:** Semi-Markov multistate model analysis of the association between monthly mean temperature and transitions of new-onset CKM multimorbidity.

Transition (From → To)	Sample size (*n*)	Number of events	Hazard ratio (HR) (per 1°C increase in monthly mean temperature)	95% confidence interval (CI)	*P*-value
No CKM → Cardiovascular-Kidney (CK) multimorbidity	14,562	62	0.937	(0.889, 0.987)	0.015
No CKM → Cardiovascular-Metabolic (CM) multimorbidity	14,562	673	0.962	(0.942, 0.981)	<0.001
No CKM → Kidney-Metabolic (KM) multimorbidity	14,562	200	0.985	(0.948, 1.023)	0.425
No CKM → Complete CKM multimorbidity	14,562	74	0.905	(0.865, 0.946)	<0.001
CK multimorbidity → Complete CKM multimorbidity	42	6	1.03	(0.879, 1.206)	0.717
CM multimorbidity → Complete CKM multimorbidity	366	24	0.982	(0.821, 1.175)	0.843
KM multimorbidity → Complete CKM multimorbidity	133	15	0.928	(0.793, 1.087)	0.356

CKM multimorbidity definition: Coexistence of at least 2 among the three disease groups (cardiovascular, kidney, and metabolic diseases) in an individual. Specific subcategories are defined as follows: Cardiovascular disease: Self-reported heart disease or stroke; Kidney disease: Self-reported chronic kidney disease; Metabolic disease: Self-reported hypertension, diabetes, or dyslipidemia; CK multimorbidity: Coexistence of cardiovascular and kidney diseases; CM multimorbidity: Coexistence of cardiovascular and metabolic diseases; KM multimorbidity: Coexistence of kidney and metabolic diseases; Complete CKM multimorbidity: Coexistence of cardiovascular, kidney, and metabolic diseases. Exposure variable: Monthly mean temperature (centered), with HR representing the change in transition risk for each 1°C increase in monthly mean temperature. Statistical methods: Semi-Markov multistate models were used to estimate HRs and 95% CIs, adjusting for age, sex, educational level, household registration type, marital status, smoking status, alcohol consumption status, and CESD depression scale score.

#### Survival mediation analysis

To quantify the mediating effects of BMI and CESD-10 score on the association between temperature exposure and incident CKM multimorbidity, a survival mediation analysis was performed. The total effect, direct effect (temperature→CKM multimorbidity), and indirect effect (temperature→mediator→CKM multimorbidity) were estimated, and the mediation proportion was calculated as (indirect effect / total effect) × 100%. The results are presented in Table 5.

**Table 5:** Survival mediation analysis of the association between temperature and new-onset CKM multimorbidity.

Mediator	Effect type	Hazard ratio (HR)	95% Confidence interval (lower–upper)	*P*-value	Mediation proportion (%)
Body Mass Index (BMI)	Total Effect	0.826	0.724–0.942	0.004	14
	Direct Effect	0.849	0.744–0.968	0.014	
	Indirect Effect	0.974	—	—	
CESD Depression Scale	Total Effect	0.858	0.753–0.979	0.023	7.6
	Direct Effect	0.849	0.744–0.968	0.014	
	Indirect Effect	1.012	—	—	

#### Subgroup analysis

**Figure 4:**
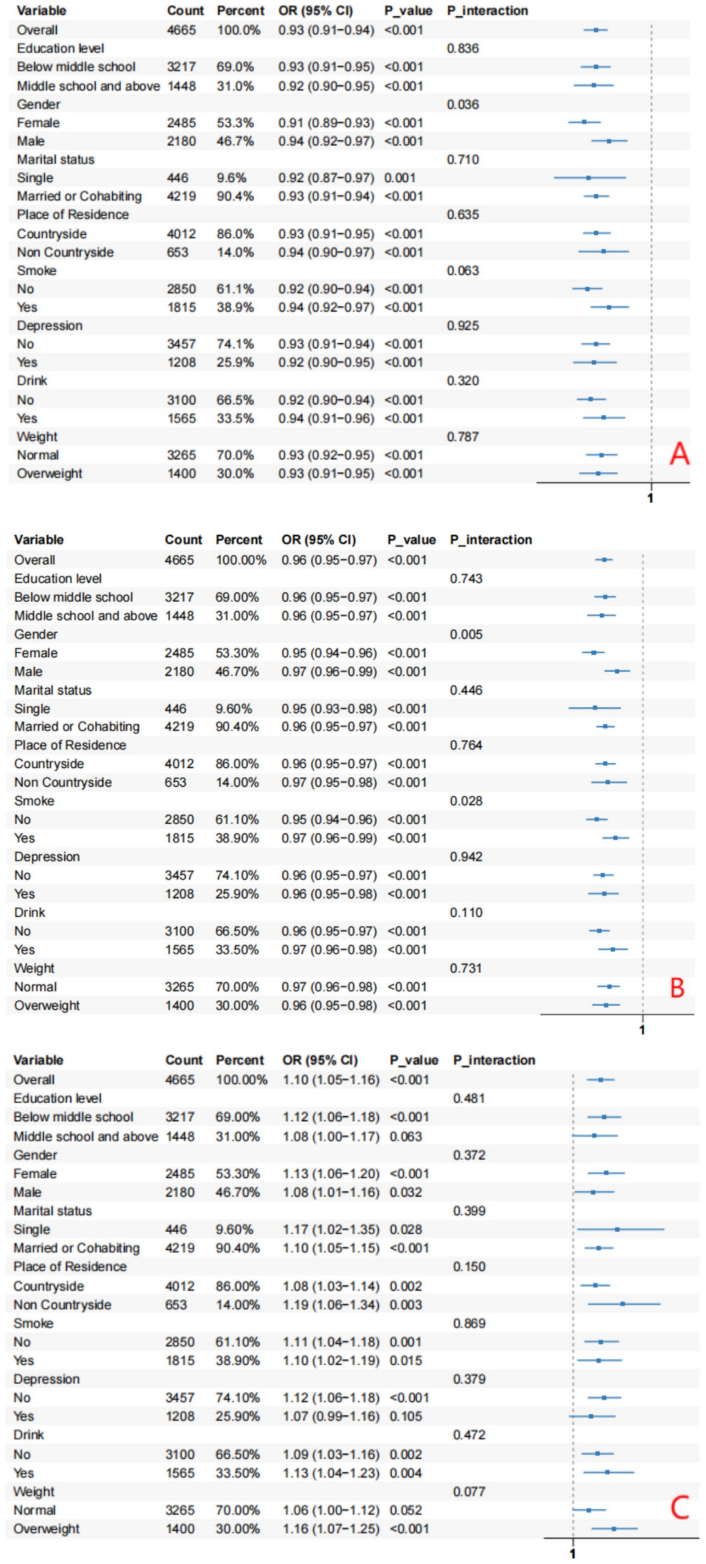
Forest plots of subgroup analyses for the association between temperature-related indicators (2011–2018) and incident CKM multimorbidity using logistic regression. **(A)** 8-year mean temperature; **(B)** 8-year minimum temperature; **(C)** Persistent extreme cold events (≥5 consecutive days). Based on baseline characteristics, including education level (below middle school/middle school and above), sex (female/male), marital status (single/married or cohabiting), place of residence (rural/urban), smoking status (never/ever smoked), alcohol consumption (less than weekly/weekly or more), depression status (CESD-10 score <12/no depressive symptoms, ≥12/with depressive symptoms), and weight status (BMI < 25 kg/m^2^/normal weight, ≥25 kg/m^2^/overweight, Asian criteria).

To investigate the heterogeneity of the association between temperature indicators and incident CKM multimorbidity, two sets of subgroup analyses were conducted:

Stratification by baseline characteristics (logistic regression-based): Analyses were stratified by education level (primary school or below/middle school or above), sex (female/male), marital status (single/married or cohabiting), place of residence (rural/urban), smoking status (never/ever smoked), alcohol consumption (less than weekly/weekly or more), depression status (CESD-10 score < 12/no symptoms, ≥12/with symptoms), and weight status (BMI < 25 kg/m^2^/normal weight, ≥25 kg/m^2^/overweight). ORs and 95% CIs were calculated for each subgroup, adjusting for all covariates. Interaction *p*-values (*P*-interaction) were computed by adding cross-product terms between exposure variables and subgroup variables to the regression models. The results are visualized in Figure 4.

Stratification by follow-up wave (Cox regression-based): Analyses were performed separately for CHARLS Wave 1 (2011), Wave 2 (2013), Wave 3 (2015), and Wave 4 (2018). Temperature was treated as a continuous variable (per 1 °C increase), and HRs and 95% CIs were estimated using Cox proportional hazards models adjusted for age, sex, BMI, and other baseline covariates. Subgroups were defined using the same baseline characteristics as the logistic regression-based analysis, and P-heterogeneity values were reported for each interaction. The results are visualized in Supplementary Figures S2–S5. All subgroup analyses were prespecified based on literature review and biological plausibility (5, 18, 21, 22). Given the exploratory nature of these analyses, no adjustment for multiple comparisons was applied; results should be interpreted as hypothesis-generating and are presented in full in the Supplementary Figures S2–S5.

### Regional heterogeneity analysis by climate zones

Participants were divided into three climate zones based on tertiles of the 8-year mean annual temperature at their residential city. Tertile cutoffs were calculated from the entire cohort (*n =* 4,665), resulting in three groups of equal size (*n =* 1,555 each): cold zone (tertile 1, lowest temperature), temperate zone (tertile 2), and warm zone (tertile 3). To formally test whether the association between persistent extreme cold events (≥5 days) and incident CKM multimorbidity differed across climate zones, we performed a multivariable logistic regression model including a product term between climate zone (categorical, with cold zone as reference) and the number of extreme cold events (continuous), adjusting for all covariates listed in Model 3 of Table 2. The *p*-value for the interaction term was reported. Additionally, we estimated the zone-specific ORs of extreme cold events by extracting the corresponding linear combinations of coefficients from the same model. Results are presented in Supplementary Table S5. Participants were resident in approximately 150 counties/districts across 28 provinces of mainland China, spanning a wide range of climatic conditions from cold northern to warm southern regions [detailed county list available in the CHARLS baseline documentation (26)].

### Quality control

*Data cleaning*: a complete case analysis was adopted for all study variables. Participants with missing data on any key variable (including temperature exposure, CKM outcome status, and covariates) were excluded from the final cohort to ensure data integrity. Outliers in meteorological data (values outside the range of mean ± 3 SD) were excluded after verification with the CMA.

*Software packages*: the following R packages were used for specific analyses: survival for Cox regression and Kaplan–Meier survival analysis, mstate for multistate models, mediation for survival mediation analysis, RMS for restricted cubic spline analysis, and forestplot for forest plot visualization.

## Results

### Study population

(1) As shown in Figure 1, a total of 25,586 participants were recruited from the baseline CHARLS survey; after applying exclusion criteria (age <45 years, missing key data, and prevalent CKM multimorbidity at baseline), the final analytic cohort included 4,665 middle-aged and older adults with a median follow-up duration of 7 years. (2) During follow-up, 804 (17.23%) participants developed incident CKM multimorbidity, while 3,861 (82.77%) remained free of the outcome.

### Sensitivity analysis for selection Bias and missing data

A comparison of baseline characteristics between the included analytic cohort (*n =* 4,665) and the participants excluded due to missing data (*n =* 20,921) is presented in Supplementary Table S2. Despite a high exclusion rate of 81.8%, all observed differences were minimal, with standardized mean differences (*SMD*) for all variables below the threshold of 0.1. The largest, albeit still negligible, differences were observed for age (*SMD* = 0.083), household registration type (*SMD* = 0.072), and depressive symptom scores (*SMD* = 0.046). These results indicate that the potential for selection bias stemming from the exclusion of participants with incomplete data is negligible.

To address the updated CHARLS 2020 data, we expanded the follow-up scope to 2011–2020 and re-screened all participants under consistent eligibility criteria. Due to widespread missing BMI values in the later survey waves, the extended sensitivity cohort included only 1,300 participants, with 46 cases of incident CKM multimorbidity. Detailed baseline comparisons between participants with and without CKM multimorbidity in this extended sample are shown in Supplementary Table S3. Despite comparable population profiles, the limited sample size and outcome events restricted statistical power for long-term effect estimation.

### Baseline characteristics

(1) As summarized in Table 1, participants who developed CKM multimorbidity were significantly older (median age: 59.00 vs. 57.00 years, *P <* 0.001), had a higher proportion of urban household registration (19.28% vs. 12.90%, *P <* 0.001), higher baseline CESD-10 scores (median: 8.00 vs. 7.00, *P <* 0.001), and higher BMI (median: 23.94 vs. 22.84 kg/m^2^, *P <* 0.001) compared with those who did not. (2) Regarding temperature-related indicators, they were exposed to significantly lower 8-year mean annual temperature (median: 15.74 vs. 16.88 °C, *P <* 0.001), lower minimum annual temperature (median: −7.68 vs. –5.85 °C, *P <* 0.001), and more persistent extreme cold events (≥5 days; median: 11.00 vs. 10.00, *P <* 0.001) during follow-up. (3) No significant between-group differences were observed in sex, educational level, marital status, smoking status, or alcohol consumption (all *p* > 0.05).

### Association between temperature exposure and incident CKM multimorbidity

**Figure 5:**
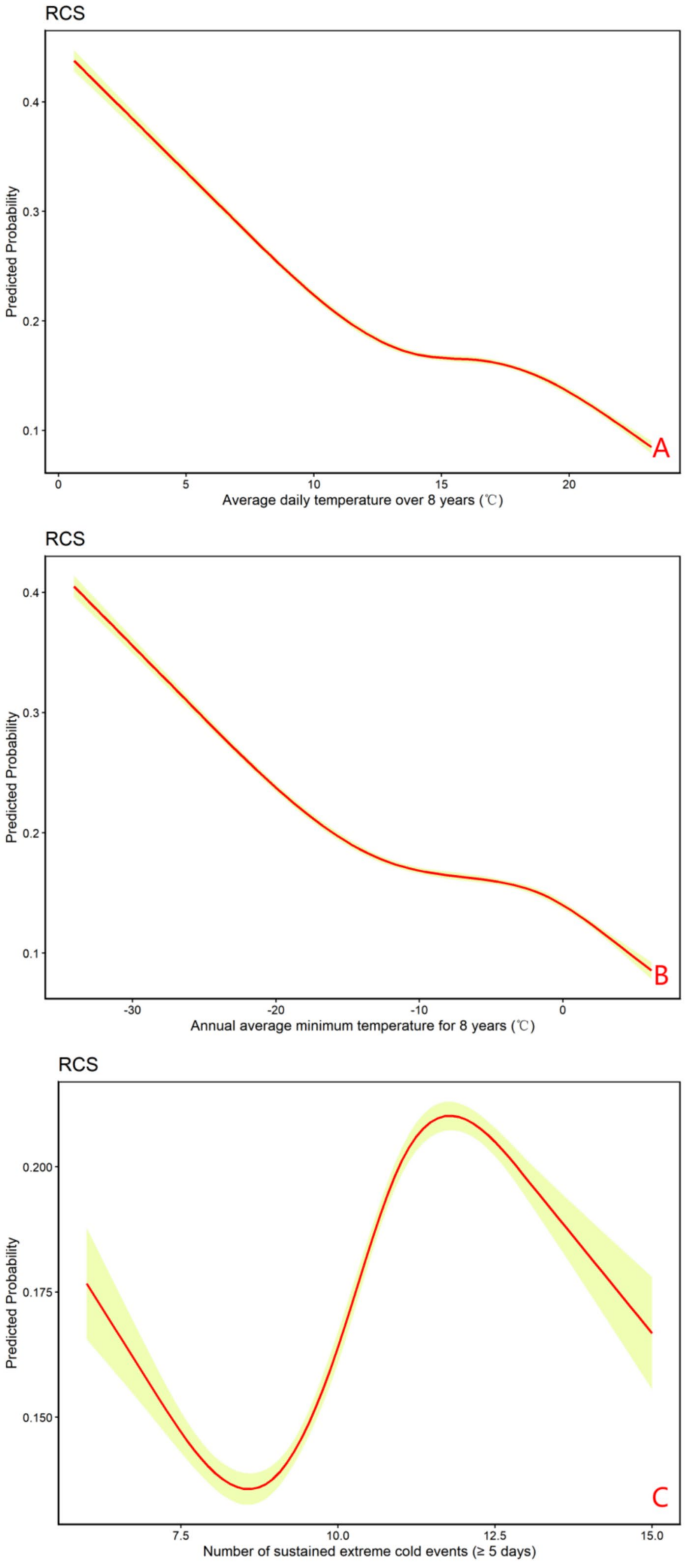
Restricted cubic spline (RCS) logistic regression of 8-year temperature metrics and incident CKM multimorbidity. **(A)** Mean temperature; **(B)** minimum temperature; **(C)** persistent extreme cold events (≥5 days). All models adjusted for age, sex, BMI, education, household registration, marital status, smoking, alcohol consumption, and CESD score. Reference values set at the 5th percentile for Wcontinuous temperatures **(A,B)** or 0 events **(C)**. Shaded areas represent 95% CIs.

(1) *Logistic regression analysis*: Table 2 shows that after full adjustment for all covariates, every 1 °C increase in 8-year mean temperature decreased the odds of incident CKM multimorbidity by 7.4% (OR = 0.926, 95% CI: 0.911–0.942, *P <* 0.001), with participants in the highest quartile (Q4: 19.70 °C) having a 56.8% lower odds than those in the lowest quartile (Q1: 12.89 °C, OR = 0.432, 95% CI: 0.343–0.544, *P* for trend = 0.01); every 1 °C increase in 8-year minimum temperature reduced the odds by 3.8% (OR = 0.962, 95% CI: 0.953–0.970, *P <* 0.001), and participants in Q4 (1.39 °C) had a 55.4% lower odds than Q1 (−11.25 °C, OR = 0.446, 95% CI: 0.354–0.561, *P* for trend = 0.025); every additional persistent extreme cold event (≥5 days) increased the odds by 10.0% (OR = 1.100, 95% CI: 1.050–1.154, *P <* 0.001), and participants in Q4 (12.0 events) had a 38.3% higher odds than Q1 (9.0 events, OR = 1.383, 95% CI: 1.111–1.721, *P* for trend = 0.004). (2) *Restricted cubic spline (RCS) analysis*: Figure 5 illustrates the non-linear dose–response relationship, where Panels A and B show a linear inverse association between 8-year mean/minimum temperature and CKM multimorbidity risk (risk decreasing steadily with increasing temperature), while Panel C demonstrates a U-shaped association between persistent extreme cold events (≥5 days) and risk, with the lowest risk at approximately 10 events.

### Survival analysis

(1) *Cox proportional hazards regression*: Table 3 indicates that each 1 °C increase in monthly mean temperature was associated with a 3.1% reduction in the hazard of incident CKM multimorbidity (HR = 0.9692, 95% CI: 0.9568–0.9817, *P <* 0.001) after adjusting for all covariates, and higher baseline CESD-10 scores (HR = 1.0523, 95% CI: 1.0383–1.0665, *P <* 0.001) and older age (HR = 1.0157, 95% CI: 1.0035–1.0280, *p* = 0.0117) were independent risk factors. (2) *Kaplan–Meier survival analysis*: As shown in Figure 2, the incidence-free survival rate of CKM multimorbidity differed significantly across monthly mean temperature groups (log-rank test, *P <* 0.0001), with the highest survival probability in the highest temperature group and the lowest in the lowest temperature group.

### Multistate model analysis

(1) Table 4 shows that higher monthly mean temperature reduced the risk of transitioning from no CKM to partial or complete CKM multimorbidity: No CKM → CK multimorbidity (HR = 0.937, 95% CI: 0.889–0.987, *p* = 0.015), No CKM → CM multimorbidity (HR = 0.962, 95% CI: 0.942–0.981, *P <* 0.001), and No CKM → Complete CKM multimorbidity (HR = 0.905, 95% CI: 0.865–0.946, *P <* 0.001), while no significant association was found for transitions from partial to complete CKM multimorbidity (all *p* > 0.05). (2) Figure 3 demonstrates that the transition probability to complete CKM multimorbidity (State 4) increased most rapidly over follow-up, followed by transitions to CM multimorbidity (State 2), indicating CM multimorbidity as the most common intermediate state.

### Survival mediation analysis

Table 5 presents an exploratory mediating assessment of relevant pathways linking temperature exposure to incident CKM multimorbidity. An exploratory mediation proportion of 14% is observed for BMI in the temperature-CKM association, while such result requires cautious interpretation given the substantial missing data of BMI indicators and self-reported disease outcomes. The indirect effect of the CESD-10 score remains non-significant (HR = 1.012, 95% CI: 0.987–1.038, *p* = 0.34). After adjustment for the two potential mediators, the direct association between temperature and CKM multimorbidity remains statistically significant (HR = 0.849, 95% CI: 0.744–0.968, *p* = 0.014), indicating independent direct effects of temperature on CKM multimorbidity risk. The non-significant mediating capacity of CESD-10 may be attributable to insufficient statistical power or complex confounding interactions, and further validation based on high-quality and complete datasets is warranted.

### Subgroup analysis

Subgroup analyses (Figure 4; Supplementary Figures S2–S5) showed that the inverse association between temperature and CKM multimorbidity was consistent across most subgroups, with significant heterogeneity only for place of residence (urban vs. rural, *P*-interaction < 0.05) and depression status (CESD-10 < 12 vs. ≥ 12, *P*-interaction < 0.05), indicating stronger protective effects of higher temperature in urban residents and those with depressive symptoms. In contrast, the positive association between persistent extreme cold events and CKM risk was consistent across all subgroups (all *P*-interaction > 0.05). Detailed results for each wave and subgroup are presented in Supplementary Figures S2–S5.

### Regional heterogeneity analysis

Stratified RCS analyses (Supplementary Figure S1) revealed significant regional heterogeneity in the association between persistent extreme cold events (≥5 days) and incident CKM multimorbidity: in warm regions, the association presented a significant non-linear pattern, with a statistically significant negative linear term (OR = 0.63, 95% CI: 0.41–0.97, *p* = 0.04), statistically significant positive first-order interaction term (OR = 5.82, 95% CI: 1.07–31.68, *p* = 0.04), and a negative quadratic term that did not reach statistical significance at the *α* = 0.05 level (OR = 0.00, 95% CI: 0.00–0.92, *p* = 0.06), and the core risk factors for CKM multimorbidity in this region included age, CESD-10 score, and baseline BMI; in cold regions, the linear term of the association showed a near-significant positive trend (OR = 1.56, *p* = 0.09), with no statistically significant effects observed in RCS interaction terms, and the core risk factors were age, CESD-10 score, and household registration type (OR = 1.87, *p <* 0.001); In temperate regions (central cities), RCS analyses showed no statistically significant association between the frequency of persistent extreme cold events and CKM multimorbidity risk (linear term: OR = 1.24, 95% CI: 0.87–1.76, *p* = 0.23; first-order interaction term: OR = 2.15, 95% CI: 0.92–5.03, *p* = 0.08; quadratic term: OR = 0.02, 95% CI: 0.00–1.35, *p* = 0.09), and household registration type showed the strongest risk effect (OR = 1.87, *p <* 0.001) among all covariates.

Formal interaction testing confirmed that the effect of persistent extreme cold events on CKM risk varied significantly between the temperate zone and the cold zone (*P* for interaction *=* 0.045), whereas no significant interaction was observed for the warm zone (*p* = 0.818). As shown in Supplementary Table S5, each additional extreme cold event was associated with a 15.6% higher odds of CKM multimorbidity in the temperate zone (OR = 1.156, 95% CI: 1.056–1.265, *p =* 0.002), but not in the cold (OR = 1.027, *p* = 0.533) or warm zone (OR = 1.043, *p* = 0.428).

## Discussion

In this CHARLS-based prospective cohort study of 4,665 middle-aged and older Chinese adults free of CKM multimorbidity at baseline, we investigated associations between temperature-related indicators and incident CKM multimorbidity using a comprehensive analytical framework including logistic regression, Cox models, semi-Markov multistate models, restricted cubic splines, and formal interaction tests. Over a median follow-up of 7 years, 804 participants (17.23%) developed incident CKM multimorbidity. Our key findings are as follows: (1) long-term higher annual mean and minimum temperatures were associated with a lower risk of CKM multimorbidity (per 1 °C increase: OR = 0.926 and 0.962, respectively); (2) each additional persistent extreme cold event (≥5 consecutive days) was associated with a 10.0% higher odds (OR = 1.100); (3) higher monthly mean temperature reduced the transition risk from no CKM to partial and complete CKM but not from partial to complete CKM; and (4) the effect of extreme cold events varied significantly by climate zone, with a formal interaction test confirming a significant interaction for the temperate zone (*p* = 0.045) but not for the warm zone (*p* = 0.818). Below we discuss these findings in the context of biological mechanisms, previous literature, and public health implications.

### Long-term ambient temperature and incident CKM multimorbidity

The inverse association between long-term ambient temperature and CKM multimorbidity risk is consistent with established biological evidence (33–35). Cold exposure activates the renin-angiotensin-aldosterone system (RAAS), induces peripheral vasoconstriction, increases blood pressure, and impairs endothelial function, thereby elevating the risk of cardiovascular disease and hypertension-core components of CKM multimorbidity (34, 35). For renal health, cold exposure reduces renal perfusion and may accelerate chronic kidney disease progression (11, 36); metabolically, it alters energy homeostasis, promotes obesity, and impairs insulin sensitivity, which are key drivers of type 2 diabetes and dyslipidemia (37, 38). Our study extends these findings by demonstrating that the adverse effects of low temperature are not limited to individual diseases but also manifest as the synergistic syndrome of CKM multimorbidity. Moreover, the restricted cubic spline analysis revealed a linear inverse dose–response relationship for mean and minimum temperature (Figures 5A,B), indicating a continuous protective effect of higher temperature without evidence of a threshold. This is supported by a recent global study based on CHARLS which found that extreme temperature events are associated with higher multimorbidity risk among older adults (19).

### Dynamic CKM transitions: evidence from the semi-Markov multistate model

The semi-Markov multistate model applied in this study captures transition processes between different CKM states, providing a unique perspective beyond traditional survival analysis that focuses only on the occurrence of the outcome (39). Our results showed that higher monthly mean temperature significantly reduced the hazard of transitioning from no CKM to CK (HR = 0.937), to CM (HR = 0.962), and directly to complete CKM (HR = 0.905), with the strongest protective effect for the direct transition to complete CKM (Table 4). In contrast, no significant associations were observed for transitions from partial CKM states (CK, CM, or KM) to complete CKM. These findings suggest that temperature exposure primarily influences the early stages of CKM development-before any component disease appears-rather than accelerating progression once a single condition is established. This has important public health implications: interventions aimed at reducing cold exposure (e.g., home heating, warm clothing, community warming centers) may be most effective when targeted at disease-free individuals. Additionally, CM multimorbidity was the most common intermediate state, and the transition probability to complete CKM increased most rapidly (Figure 3). This aligns with the view that cardiovascular and metabolic diseases share common pathophysiological mechanisms such as chronic inflammation and oxidative stress, and their co-occurrence may accelerate renal involvement, eventually leading to complete CKM multimorbidity (40–42). To our knowledge, this is the first study to apply a semi-Markov multistate model to explore temperature-driven CKM progression.

### Regional heterogeneity and formal interaction testing

We further examined regional heterogeneity by dividing participants into three climate zones based on tertiles of the 8-year mean temperature (cold, temperate, warm; each *n =* 1,555). Formal interaction testing was performed using a logistic regression model that included a product term between climate zone (categorical, with cold zone as reference) and the number of persistent extreme cold events (continuous), adjusting for all covariates. As shown in Supplementary Table S5, the interaction was statistically significant for the temperate zone compared with the cold zone (*P* for interaction *=* 0.045), whereas the warm zone did not differ significantly from the cold zone (*p* = 0.818). The zone-specific odds ratios revealed that each additional extreme cold event increased CKM odds by 15.6% in the temperate zone (OR = 1.156, 95% CI: 1.056–1.265), but not in the cold zone (OR = 1.027, *p* = 0.533) or the warm zone (OR = 1.043, *p* = 0.428).

These findings are consistent with the “adaptation hypothesis.” Residents in cold regions may have developed physiological (e.g., enhanced non-shivering thermogenesis) and behavioural (e.g., central heating, heavy clothing) adaptations that mitigate the impact of extreme cold events (24, 25). Warm-zone residents rarely experience such events, so when they occur, they may be short-lived and less physiologically disruptive. Temperate-zone populations encounter extreme cold events with moderate frequency but may lack full adaptive mechanisms, making them particularly vulnerable. This interpretation is supported by a nationwide Chinese study showing weaker temperature-mortality associations in colder cities and a recent study on frailty and diabetes (24, 34). However, these are post-hoc hypotheses; individual-level measures of adaptation (e.g., heating availability, clothing insulation) are needed to confirm the pathways. In the stratified RCS analyses (Supplementary Table S1), the warm region showed a non-linear pattern with a significant negative linear term (OR = 0.63, *p* = 0.04)-which may reflect a hormetic effect where moderate cold stress temporarily improves metabolic efficiency-while the cold and temperate regions showed null or borderline associations (43). Notably, household registration type (urban vs. rural) was a strong risk factor in cold and temperate zones (OR = 1.87, *p <* 0.001), suggesting that urban–rural differences in lifestyle and healthcare access modify the climate-health association, as previously reported (21).

### Exploratory mediation analysis

Our survival mediation analysis (Table 5) suggested that BMI may partially mediate the temperature-CKM association, with a mediation proportion of 14%. Biologically, chronic cold exposure can increase energy intake and reduce energy expenditure, leading to weight gain (44, 45), and obesity is a well-established risk factor for all three CKM components. However, due to the high proportion of missing BMI data (46.72%) and the self-reported nature of outcome diagnoses, this finding should be interpreted as exploratory and hypothesis-generating rather than confirmatory. The direct effect of temperature on CKM risk remained significant after adjusting for BMI (HR = 0.849, *p* = 0.014), indicating that other pathways-such as RAAS activation, direct vascular injury, or inflammatory responses-also contribute (22, 46–48). Depressive symptoms (CESD-10) showed no significant indirect effect, possibly due to limited statistical power or the complex interplay between temperature, mood, and chronic disease.

### Subgroup findings and public health implications

Subgroup analyses (Figure 4) showed that the inverse association between temperature and CKM multimorbidity was more pronounced in urban residents and those with depressive symptoms. For urban residents, better living conditions (e.g., centralized heating) may enhance sensitivity to temperature changes, making the protective effect of higher temperature more obvious. For individuals with depressive symptoms, who often have unhealthy lifestyles and weakened immune function, they may be more vulnerable to cold exposure; thus, the benefit of higher temperature is amplified. In contrast, the positive association between persistent extreme cold events and CKM risk was consistent across all subgroups, indicating that extreme cold events are a universal risk factor. The stable inverse association across all follow-up waves (2011–2018) (Supplementary Figures S2–S5) further strengthens the reliability of our findings.

Based on these results, we propose targeted interventions to mitigate CKM multimorbidity risk in vulnerable populations. For cold and temperate regions (particularly northern and central China), interventions should include: (1) enhanced home insulation programs and affordable heating subsidies for low-income older adults, especially those with rural household registration who showed heightened vulnerability; (2) early-warning systems for persistent extreme cold events (≥5 days) coupled with community warming centers and health monitoring for adults aged 50 + with pre-existing metabolic conditions; and (3) integrated weight management programs in cold regions to address the BMI-mediated pathway. For urban residents and those with depressive symptoms, who demonstrated stronger protective effects from higher temperatures, interventions could focus on creating temperature-controlled community spaces and mental health support during cold seasons.

### Limitations

This study has several limitations. First, incident CKM multimorbidity was based on self-reported diagnoses without objective validation, potentially introducing recall bias. Second, we did not adjust for important unmeasured confounders, including ambient air pollution, physical activity, and dietary habits. Although we used residential location and household registration as crude proxies, residual confounding cannot be fully ruled out. Future studies should integrate high-resolution air pollution data and detailed lifestyle questionnaires. Third, the original sample had a high overall missing rate (81.8%), with BMI missing 46.72%; this limits representativeness and generalizability, especially for the exploratory mediation analysis. Fourth, genetic susceptibility factors were not considered, and the mechanisms underlying regional heterogeneity remain unclear. Fifth, mediation analyses are exploratory only; the substantial missing BMI data and self-reported outcomes preclude robust causal inference, and definitive mediating effects cannot be confirmed without complete, clinically validated datasets.

## Conclusion

This study confirms that long-term low temperature and persistent extreme cold events increase incident CKM multimorbidity risk in middle-aged and older adults, with effects on early disease transitions and regional heterogeneity. BMI mediates part of the association. These findings fill evidence gaps and provide a basis for targeted prevention strategies (e.g., enhanced protection in cold regions) to mitigate CKM burden amid ageing.

## Data Availability

The data used in this study are derived from two publicly available and ethically approved sources: China Health and Retirement Longitudinal Study (CHARLS) data (2011–2018): The dataset is publicly accessible via the official CHARLS platform (https://charls.pku.edu.cn/). Researchers may obtain the data by submitting a formal application and complying with the CHARLS Data Usage Agreement, which includes provisions for data privacy protection and academic attribution. Relevant citations for the CHARLS dataset are: Zhao Y, Hu Y, Smith JP, et al. Cohort Profile: The China Health and Retirement Longitudinal Study (CHARLS). Int J Epidemiol. 2014;43(1):61–68; Zhao Y, Strauss J, Yang G, et al. China Health and Retirement Longitudinal Study: 2011-2012 National Baseline User’s Guide. National School of Development, Peking University, 2013. Meteorological data (2011–2018): Daily temperature data (maximum, minimum, mean) were acquired from the China Meteorological Administration (CMA) National Surface Meteorological Station Network (http://data.cma.cn/), a public meteorological data service platform. Data were matched to participants via geocoded residential information (maximum matching distance: 50 km) to ensure accuracy. The processed datasets (including linked temperature-health outcome data, de-identified to protect participant privacy) and statistical analysis code (R software, version 4.3.1) used in the current study are available from the corresponding author (JianChao Li: 18539989191@163.com) upon reasonable request. All data sharing complies with the ethical approval requirements of the CHARLS study (Ethics Review Committee of Peking University, IRB00001052-11015) and relevant national regulations on data management.
