# Supplementary material for: Temperature-related indicators and risk of incident cardiovascular-kidney-metabolic multimorbidity in middle-aged and older adults: a longitudinal cohort study from CHARLS
Source: Front Public Health. 2026 Jun 25;14:1823025. doi: 10.3389/fpubh.2026.1823025 (PMC13346223; doi:10.3389/fpubh.2026.1823025)
Supplement: Supplementary file 1 [file Data_Sheet_1.docx]

**Table S1 Missing Data Patterns for Key Variables in the Initial Sample (n=25,586)**

| **Variable** | **Number of missing values** | **Missing rate**  **(%)** |
| --- | --- | --- |
| Age at Wave 1, years | 8053 | 31.47 |
| Sex | 8 | 0.03 |
| Smoking status at Wave 1 | 8027 | 31.37 |
| Body mass index (BMI) at Wave 1 | 11955 | 46.72 |
| Household registration type at Wave 1 | 7913 | 30.93 |
| Marital status at Wave 1 (including cohabitation) | 7890 | 30.84 |
| CESD-10 score at Wave 1 | 9549 | 37.32 |
| Alcohol consumption in past year at Wave 1 | 8034 | 31.40 |
| Educational level (detailed classification) | 44 | 0.17 |

**Table S2 Comparison of Baseline Characteristics Between Included and Excluded Participants**

| **Variable** | **Included cohort**  **(n=4665)** | **Excluded participants**  **(n=20921)** | ***P*value** | **SMD** |
| --- | --- | --- | --- | --- |
| Demographic Characteristics |  |  |  |  |
| Age at Wave 1, years (median, IQR) | 57.00 (51.00, 63.00) | 55.22 (50.00, 62.00) | <0.001 | 0.083 |
| Educational level, n (%) |  |  | 0.437 | 0.005 |
| Primary school or below | 3217 (68.96%) | 14302 (68.36%) |  |  |
| Middle school or above | 1448 (31.04%) | 6619 (31.64%) |  |  |
| Sex, n (%) |  |  | 0.027 | 0.014 |
| Male | 2180 (46.73%) | 10154 (48.53%) |  |  |
| Female | 2485 (53.27%) | 10767 (51.47%) |  |  |
| Marital status at Wave 1, n (%) |  |  | 0.043 | 0.013 |
| Unmarried/divorced/widowed | 446 (9.56%) | 1803 (8.62%) |  |  |
| Married/cohabiting | 4219 (90.44%) | 19118 (91.38%) |  |  |
| Household registration type at Wave 1, n (%) |  |  | <0.001 | 0.072 |
| Rural | 4012 (86.00%) | 16423 (78.50%) |  |  |
| Urban | 653 (14.00%) | 4498 (21.50%) |  |  |
| Lifestyle and Mental Health |  |  |  |  |
| Smoking status at Wave 1, n (%) |  |  | 0.289 | 0.007 |
| Never smoked | 2850 (61.09%) | 12603 (60.24%) |  |  |
| Ever smoked | 1815 (38.91%) | 8318 (39.76%) |  |  |
| Alcohol consumption in past year at Wave 1, n (%) |  |  | <0.001 | 0.026 |
| No | 3100 (66.45%) | 14567 (69.63%) |  |  |
| Yes | 1565 (33.55%) | 6354 (30.37%) |  |  |
| CESD10 score at Wave 1 (median, IQR) | 7.00 (3.00, 12.00) | 7.59 (5.00, 10.24) | <0.001 | 0.046 |
| Baseline Clinical Indicator |  |  |  |  |
| Body mass index (BMI) at Wave 1, kg/m² (median, IQR) | 23.01 (20.75, 25.62) | 24.07 (21.91, 25.70) | <0.001 | 0.030 |

Continuous variables are presented as median (interquartile range, IQR) due to non-normal distribution; categorical variables are presented as frequency (percentage);Statistical analyses: Categorical variables were compared using the χ² test; continuous variables were compared using the Mann-Whitney U test;CESD-10: 10-item Center for Epidemiologic Studies Depression Scale;A two-tailed P<0.05 was considered statistically significant.

**Table S3 Baseline Characteristics of the 2011–2020 Sensitivity Cohort, Stratified by Incident CKM Multimorbidity**

| **Variable** | **No CKM multimorbidity (n=1254)** | **With CKM multimorbidity (n=46)** | ***P*-value** |
| --- | --- | --- | --- |
| Demographic Characteristics |  |  |  |
| Age at Wave 1, years, median (IQR) | 56.00 (50.00, 61.00) | 59.00 (54.00, 64.00) | 0.006 |
| Educational level, n (%) |  |  | 0.424 |
| Primary school or below | 1079 (86.04%) | 42 (91.30%) |  |
| Middle school or above | 175 (13.96%) | 4 (8.70%) |  |
| Sex, n (%) |  |  | – |
| Male | – | – | – |
| Female | – | – | – |
| Marital status at Wave 1, n (%) |  |  | 0.739 |
| Unmarried/divorced/widowed | 143 (11.40%) | 4 (8.70%) |  |
| Married/cohabiting | 1111 (88.60%) | 42 (91.30%) |  |
| Household registration type at Wave 1, n (%) |  |  | 0.706 |
| Rural | 1075 (85.73%) | 38 (82.61%) |  |
| Urban | 179 (14.27%) | 8 (17.39%) |  |
| Lifestyle and Mental Health |  |  |  |
| Smoking status at Wave 1, n (%) |  |  | 0.023 |
| Never smoked | 1127 (89.87%) | 36 (78.26%) |  |
| Ever smoked | 127 (10.13%) | 10 (21.74%) |  |
| Alcohol consumption in past year at Wave 1, n (%) |  |  | <0.001 |
| No | 730 (58.21%) | 14 (30.43%) |  |
| Yes | 524 (41.79%) | 32 (69.57%) |  |
| CESD-10 score at Wave 1, median (IQR) | 5.00 (3.00, 10.00) | 10.00 (6.25, 14.00) | <0.001 |
| Baseline Clinical Indicator |  |  |  |
| Stroke diagnosis at Wave 1, n (%) |  |  | <0.001 |
| No | 1254 (100.00%) | 44 (95.65%) |  |
| Yes | 0 (0.00%) | 2 (4.35%) |  |
| Temperature-Related Indicators (2011–2020) |  |  |  |
| Mean annual temperature, °C, median (IQR) | 16.73 (13.93, 18.45) | 16.94 (13.95, 18.44) | 0.944 |
| Minimum annual temperature, °C, median (IQR) | -5.93 (-13.24, -1.05) | -4.54 (-12.61, -0.59) | 0.647 |
| Maximum annual temperature, °C, median (IQR) | 31.54 (30.84, 32.81) | 32.11 (31.07, 33.02) | 0.175 |
| Persistent extreme cold events, n, median (IQR) |  |  |  |
| ≥2 days | 30.00 (26.00, 32.00) | 28.50 (25.00, 32.00) | 0.035 |
| ≥3 days | 20.00 (18.00, 21.00) | 19.00 (16.25, 21.00) | 0.014 |
| ≥5 days | 10.00 (9.00, 11.00) | 10.00 (9.00, 11.00) | 0.581 |
| ≥7 days | 5.00 (4.00, 6.00) | 5.00 (5.00, 7.00) | 0.025 |
| Persistent extreme heat events, n, median (IQR) |  |  |  |
| ≥2 days | 28.00 (24.00, 34.00) | 29.00 (25.00, 33.00) | 0.556 |
| ≥3 days | 18.00 (15.00, 21.00) | 18.00 (16.00, 20.75) | 0.946 |
| ≥5 days | 8.00 (6.00, 9.00) | 8.00 (6.00, 9.00) | 0.479 |
| ≥7 days | 4.00 (3.00, 6.00) | 4.00 (3.00, 6.00) | 0.324 |
| Seasonal mean temperature, °C, median (IQR) |  |  |  |
| Q1 (Jan–Mar): Mean | 7.62 (2.80, 10.30) | 8.13 (2.95, 10.21) | 0.983 |
| Q1 (Jan–Mar): Minimum mean | -1.35 (-7.41, 2.96) | 0.17 (-6.78, 2.78) | 0.881 |
| Q1 (Jan–Mar): Maximum mean | 18.54 (16.88, 20.29) | 18.98 (16.72, 20.26) | 0.966 |
| Q2 (Apr–Jun): Mean | 21.35 (19.61, 22.40) | 21.90 (19.83, 22.62) | 0.682 |
| Q2 (Apr–Jun): Minimum mean | 10.01 (7.11, 12.79) | 10.02 (7.74, 13.23) | 0.751 |
| Q2 (Apr–Jun): Maximum mean | 29.13 (27.02, 29.98) | 29.12 (27.96, 29.83) | 0.823 |
| Q3 (Jul–Sep): Mean | 25.11 (23.21, 26.97) | 25.60 (23.25, 26.63) | 0.852 |
| Q3 (Jul–Sep): Minimum mean | 17.60 (15.21, 19.06) | 17.92 (15.35, 18.78) | 0.922 |
| Q3 (Jul–Sep): Maximum mean | 30.89 (28.63, 31.64) | 31.10 (29.21, 31.86) | 0.283 |
| Q4 (Oct–Dec): Mean | 12.06 (7.37, 14.55) | 11.87 (7.92, 13.97) | 0.847 |
| Q4 (Oct–Dec): Minimum mean | 0.69 (-5.21, 4.59) | 1.70 (-4.57, 4.81) | 0.816 |

CKM multimorbidity: Coexistence of at least 2 conditions among cardiovascular disease, kidney disease, and metabolic disease (hypertension, diabetes, dyslipidemia) during the 2011–2020 follow-up.Continuous variables are presented as median (interquartile range, IQR) due to non-normal distribution; categorical variables are presented as frequency (percentage).Statistical analyses: Categorical variables were compared using the χ² test; continuous variables were compared using the Mann–Whitney U test.CESD-10: 10-item Center for Epidemiologic Studies Depression Scale.A two-tailed *P* < 0.05 was considered statistically significant.Extreme temperature events were defined based on local climate thresholds.

**Table S4. Schoenfeld residual tests for the proportional hazards assumption**

| **Variables** | **Chi-square** | **df** | ***P*-value** |
| --- | --- | --- | --- |
| Monthly mean temperature | 1.138 | 1 | 0.286 |
| Current age | 3.096 | 1 | 0.078 |
| Sex | 0.92 | 1 | 0.337 |
| Education level | 1.98 | 1 | 0.159 |
| Household registration type | 0.081 | 1 | 0.776 |
| Marital status | 1.927 | 1 | 0.165 |
| Smoking status | 0.176 | 1 | 0.675 |
| Drinking status | 0.265 | 1 | 0.607 |
| Global test | 12.289 | 8 | 0.139 |

Note: *P* > 0.05 for all variables and the global test, indicating the proportional hazards assumption was adequately satisfied.

**Table S5. Association between persistent extreme cold events (≥5 days) and incident CKM multimorbidity across climate zones (based on 8-year mean temperature tertiles), with interaction tests**

| **Climate zone** | **Sample size**  **(n)** | **OR per 1 event increase**  **(95% CI)** | ***P*-value** | ***P* for interaction** |
| --- | --- | --- | --- | --- |
| Cold  (Tertile 1, reference) | 1,555 | 1.027 (0.945–1.117) | 0.533 | — |
| Temperate (Tertile 2) | 1,555 | 1.156 (1.056–1.265) | 0.002 | 0.045 |
| Warm  (Tertile 3) | 1,555 | 1.043 (0.941–1.156) | 0.428 | 0.818 |

Abbreviations: CKM, cardiovascular-kidney-metabolic; OR, odds ratio; CI, confidence interval.

Adjusted for age, sex, education, marital status, household registration, smoking, alcohol consumption, CESD‑10 score, and BMI.

*P* for interaction derived from a logistic regression model including a product term between climate zone (as categorical, cold as reference) and the number of persistent extreme cold events (continuous), adjusted for the same covariates.


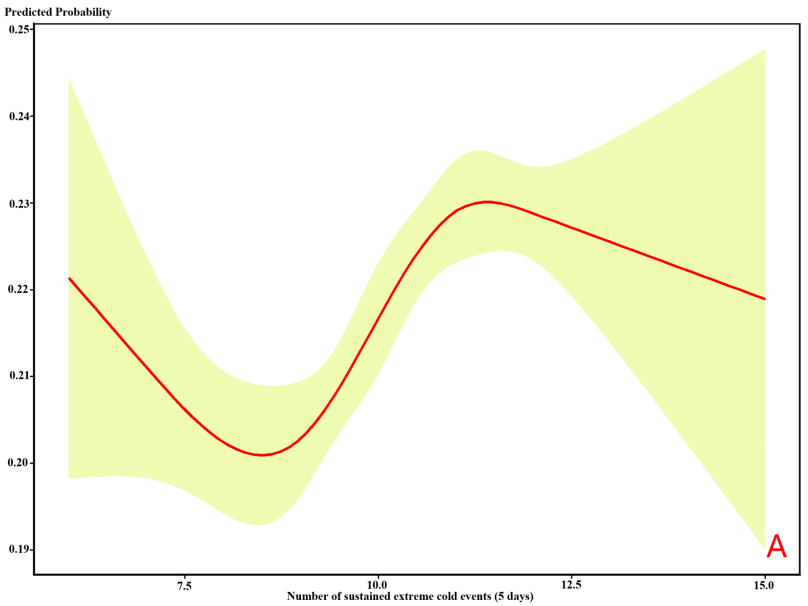


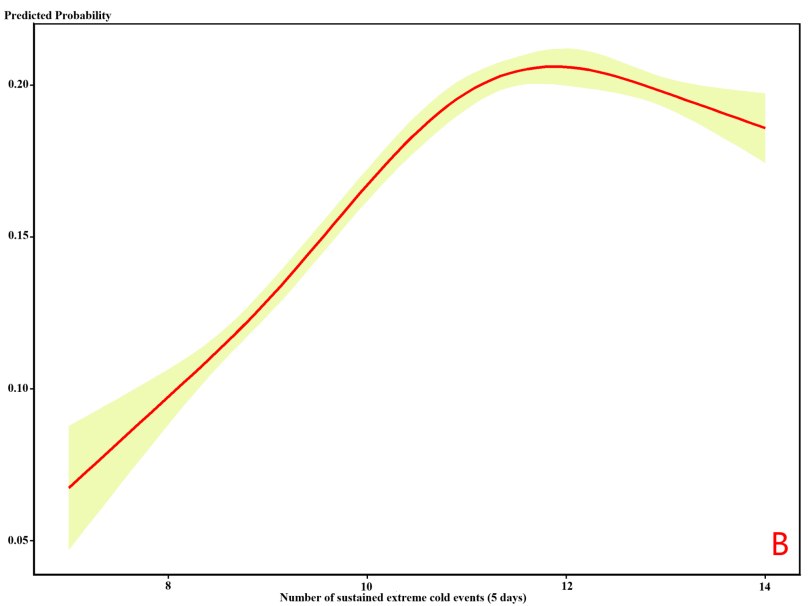

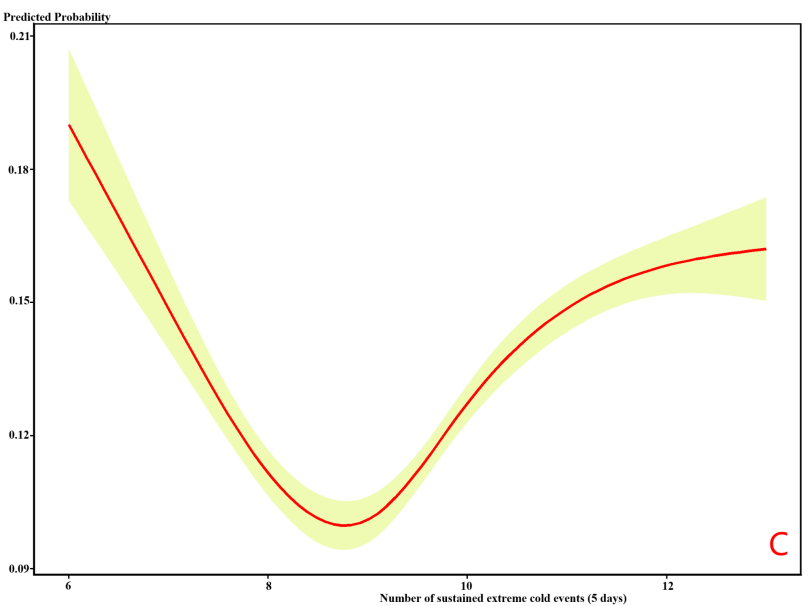


**Figure S1. Regional heterogeneity in the association between persistent extreme cold events and risk of cardiorenal metabolic syndrome (CKM)**

A.Restricted cubic spline (RCS) curve of the association between persistent extreme cold events and CKM risk in the Coldregion;

B.Restricted cubic spline (RCS) curve of the association between persistent extreme cold events and CKM risk in the Temperateregion;

C.Restricted cubic spline (RCS) curve of the association between persistent extreme cold events and CKM risk in the Warmregion.

Regional heterogeneity in the association between persistent extreme cold events and CKM risk. Restricted cubic spline (RCS) curves fitted with 3 knots (at the 10th, 50th, and 90th percentiles of the exposure distribution) for the cold (A), temperate (B), and warm (C) regions. All models adjusted for age, education, gender, marital status, household registration type, smoking status, CESD-10 score, alcohol consumption, and baseline BMI. Shaded areas represent 95% confidence intervals.


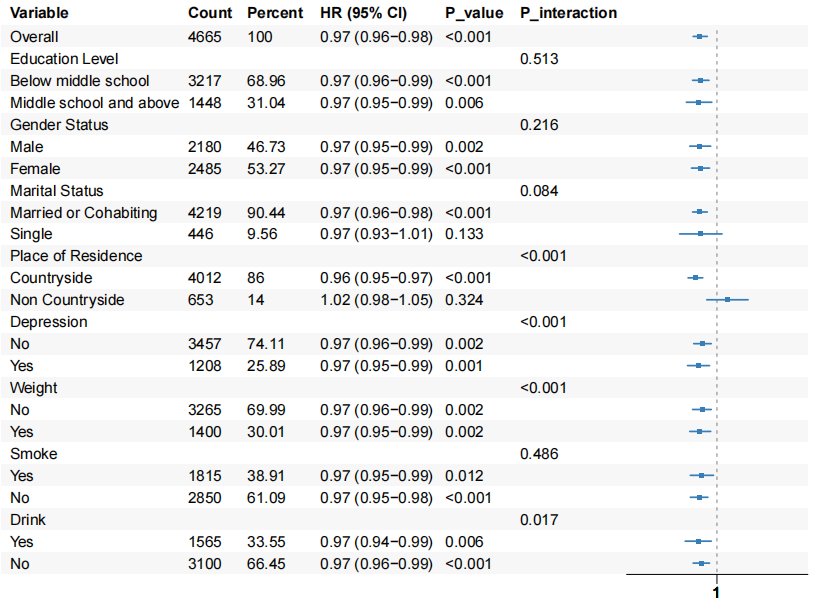


**Figure S2. Forest Plots of Cox Regression Subgroup Analyses for the Association Between Temperature and New-Onset CKM Multimorbidity in CHARLS (Wave 1, 2011)**


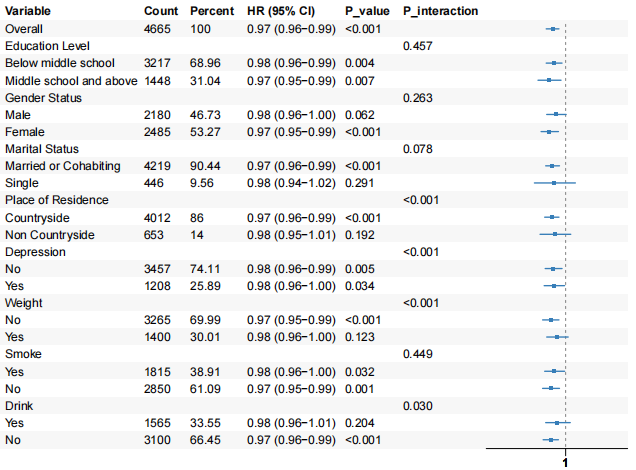


**Figure S3. Forest Plots of Cox Regression Subgroup Analyses for the Association Between Temperature and New-Onset CKM Multimorbidity in CHARLS (Wave 2, 2013)**


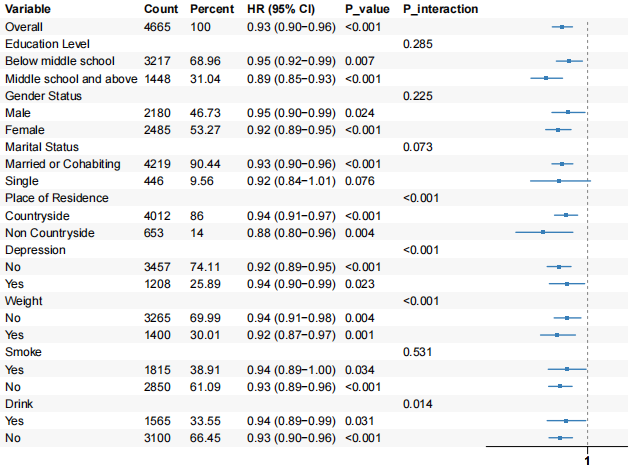


**Figure S4. Forest Plots of Cox Regression Subgroup Analyses for the Association Between Temperature and New-Onset CKM Multimorbidity in CHARLS (Wave 3, 2015)**


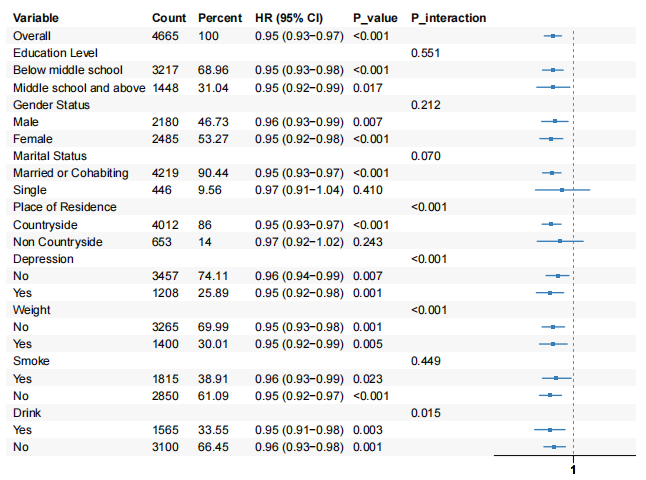


**Figure S5. Forest Plots of Cox Regression Subgroup Analyses for the Association Between Temperature and New-Onset CKM Multimorbidity in CHARLS (Wave 4, 2018)**
